# Combined endoscopic and laparoscopic surgery (CELS) for early colon cancer in high-risk patients

**DOI:** 10.1007/s00464-023-10385-3

**Published:** 2023-09-28

**Authors:** Morten F. S. Hartwig, Mustafa Bulut, Jens Ravn-Eriksen, Lasse B. Hansen, Rasmus D. Bojesen, Mads Falk Klein, Henrik L. Jakobsen, Morten Rasmussen, Bo Rud, Jens-Ole Eriksen, Susanne Eiholm, Anne-Marie K. Fiehn, Phil Quirke, Ismail Gögenur

**Affiliations:** 1 Department of Surgery, Center for Surgical Science, Zealand University Hospital Koege, Lykkebaekvej 1, 4600 Koege, Denmark; 2 Department of Surgery, Zealand University Hospital, Koege, Denmark; 3 Department of Clinical Medicine, University of Copenhagen, Copenhagen, Denmark; 4 Department of Surgery, Copenhagen University Hospital - Herlev & Gentofte Hospital, Herlev, Denmark; 5 Department of Surgery, Copenhagen University Hospital - Bispebjerg Hospital, Copenhagen, Denmark; 6 Department of Surgery, Copenhagen University Hospital - Hvidovre Hospital, Hvidovre, Denmark; 7 Department of Pathology, Zealand University Hospital, Roskilde, Denmark; 8 Pathology & Data Analytics, Leeds Institute of Medical Research at St James’s, University of Leeds, Leeds, UK

**Keywords:** Colon cancer, Early cancer, Combined endoscopy and laparoscopy

## Abstract

**Background:**

Local excision of early colon cancers could be an option in selected patients with high risk of complications and no sign of lymph node metastasis (LNM). The primary aim was to assess feasibility in high-risk patients with early colon cancer treated with Combined Endoscopic and Laparoscopic Surgery (CELS).

**Methods:**

A non-randomized prospective feasibility study including 25 patients with Performance Status score ≥ 1 and/or American Society of Anesthesiologists score ≥ 3, and clinical Union of International Cancer Control stage-1 colon cancer suitable for CELS resection. The primary outcome was failure of CELS resection, defined as either: Incomplete resection (R1/R2), local recurrence within 3 months, complication related to CELS within 30 days (Clavien–Dindo grade ≥ 3), death within 30 days or death within 90 days due to complications to surgery.

**Results:**

Fifteen patients with clinical T1 (cT1) and ten with clinical T2 (cT2) colon cancer and without suspicion of metastases were included. Failure occurred in two patients due to incomplete resections. Histopathological examination classified seven patients as having pT1, nine as pT2, six as pT3 adenocarcinomas, and three as non-invasive tumors. In three patients, the surgical strategy was changed intraoperatively to conventional colectomy due to tumor location or size. Median length of stay was 1 day. Seven patients had completion colectomy performed due to histological high-risk factors. None had LNM.

**Conclusions:**

In selected patients, CELS resection was feasible, and could spare some patients large bowel resection.

Colorectal cancer is the third most common cancer diagnosed worldwide, and accounts for almost 10% of all cancer-related deaths only outnumbered by lung cancer [1]. The only curable treatment is surgical resection. According to the American Society of Colon and Rectal Surgeons guidelines, treatment for localized colon cancer should include resection of the tumor and its lymphovascular drainage, covering a minimum of 5–7 cm of proximal and distal colon, to ensure complete resection of the tumor-bearing bowel segment and the mesocolic lymph nodes [2]. In contrast, polyp cancers (pT1) with free resection margins and no histopathological high-risk features can be safely treated with polypectomy and surveillance endoscopy. This recommendation is based on a relatively low risk of lymph node metastasis (LNM) [2, 3]. For patients with early-stage colon cancer (i.e., T1–T2), the risk of LNM is less than 20% [4, 5]. This risk and its influence on long-term oncological outcomes should be carefully considered, but may be of less importance in elderly and frail patients, that have a higher risk of complications and postoperative mortality after conventional oncological resections [6–8]. More than 33% of patients with colon cancer have WHO Performance Score (PS) 1 or 2, and these patients have a 10% and 18% risk of 1-year mortality, respectively, after elective colon cancer surgery [9].

Combined Endoscopic and Laparoscopic Surgery (CELS) was first reported in 1993, and is now an established procedure for managing difficult polyps that are not suitable for endoscopic removal alone [10–12]. The CELS procedure is a common designation for various procedures with laparoscopic or endoscopic resection combined with simultaneous intra- and extraluminal view.

Our hypothesis was that frail and comorbid patients with a high risk of adverse outcomes and early-stage colon cancer could safely be treated with a laparoscopic wedge resection guided by simultaneous endoscopic view.

The primary aim of this study was to assess feasibility and safety in high-risk patients with early colon cancer treated with CELS resection.

## Materials and methods

### Study design and participants

This was a prospective non-randomized multicenter feasibility study. The study was conducted at four regional colorectal cancer centers. The experience with CELS in the departments ranged from no experience to more than 50 procedures performed on benign tumors. To test the feasibility on various tumor locations and sizes, the aim was to include 25 patients.

The inclusion criteria were: patients above 18 years of age, strong suspicion of or biopsy-proven adenocarcinoma in the colon, clinical Union of International Cancer Control (UICC) stage-1 (cT1-2cN0cM0) tumor based on multi-detector computed tomography (CT) scan, high-risk patients defined as American Society of Anesthesiologists (ASA) score ≥ 3 and/or PS score ≥ 1. The tumor had to be assessed suitable for local resection with CELS at the multidisciplinary team conference (MDT) prior to treatment. This included that the tumor involved less than 50% of the bowel lumen at insufflation and did not involve the ileocecal valve.

Patients were excluded if they had undergone preoperative chemo- or radiotherapy, were not able to give informed consent or if a poorly differentiated component, mucinous component, or signet cell carcinoma were identified in the biopsies.

### Surgery

Before surgery, the patients underwent bowel preparation according to the department’s standard procedure. Patients were under general anesthesia, and intravenous antibiotic prophylaxis was administered. A 12 mm camera port was inserted and pneumoperitoneum was established with a pressure of 12 mmHg, and additional ports were placed according to the location of the tumor. The terminal ileum was clamped using a laparoscopic grasper and the endoscope was inserted. The tumor was located endoscopically and confirmed laparoscopically using translucent light from the endoscope and laparoscopic markings by, e.g., graspers and endoscopic tripod forceps. When the tumor was assessed suitable for local resection, the colon was mobilized according to tumor placement in order to resect the tumor area using laparoscopic staplers. For maneuvering the stapler over the tumor area, the endoscope removed all intraluminal air. The tumor was resected using the laparoscopic staplers and placed in a retrieval bag and removed through the camera port site. The resection site was viewed intra- and extra luminal to ensure a free lumen and no residual tumor. The tumor specimen was cut open alongside the stapled line in the operating room to ensure macroscopic complete resection. At two sites, the pathologists were able to perform the procedure together with the surgeons.

If CELS was not possible, the surgeons proceeded to standard colectomy. Postoperatively, patients were transferred to the surgical ward in an enhanced recovery setting and discharged according to the department’s standard procedure. The patients were referred to the outpatient clinic and informed of the histological evaluation approximately 2 weeks after surgery.

If the pathological assessment showed a microscopically radical resection (R0 resection), pT-category 1 or 2, and no histopathological risk factors, the patients were referred to a watchful waiting group.

**Fig. 1:**
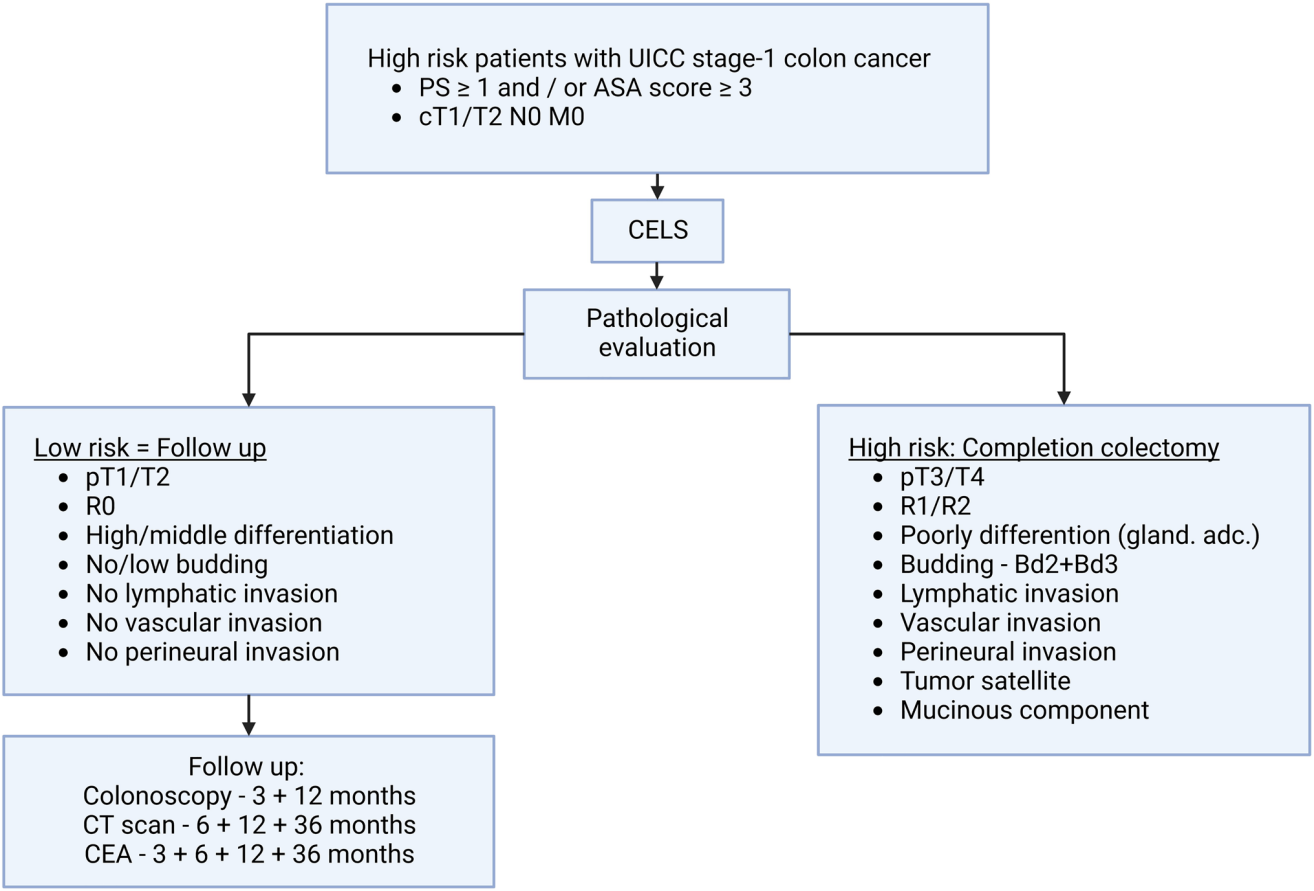
Patient selection and treatment stratification

All patients were discussed at a postoperative MDT conference, and for patients with the presence of risk factors, incomplete resection or > pT2 category, completion colectomy (standard oncological resection) was recommended. In cases where the patient was not regarded fit for completion colectomy, further treatment or follow-up recommendations were discussed with the patient. The histopathological risk factors and follow-up strategy are described in Fig. 1.

### Outcome

The primary outcome was failure after CELS resection. Failure was defined as incomplete resection (R1/R2, defined as ≤ 1 mm to circumferential resection margin or surgical resection margin), local recurrence within 3 months, complication related to CELS within 30 days (Clavien–Dindo ≥ 3 [13]), or death within 30 days of any cause or death within 90 days due to complications to surgery.

The secondary outcomes were registration of study inclusion period, number of patients converted to conventional resection, and number of patients referred to completion colectomy based on histologic risk factors. The aim of the secondary outcomes was to assess feasibility.

### Safety

An external independent safety committee consisting of two external certified colorectal surgeons and one external pathologist monitored the study endpoints. If a patient had an incomplete resection (R1/R2) or a severe complication (Clavien–Dindo ≥ 3b), the external safety committee evaluated the specimen and the treatment of the patient in regard to discussing the termination of the study. If four patients had R1/R2 resections or if three patients had local recurrence within 3 months, the study would automatically be terminated.

The primary site of this study was Zealand University Hospital, where the surgical department had 4 years of experience with CELS resection of benign tumors (+ 50 procedures). Patients not recruited at the primary site were reviewed by the MDT conference at Zealand University Hospital, including video or photo material of the endoscopic procedure, for external assessment prior to treatment.

## Results

**Fig. 2:**
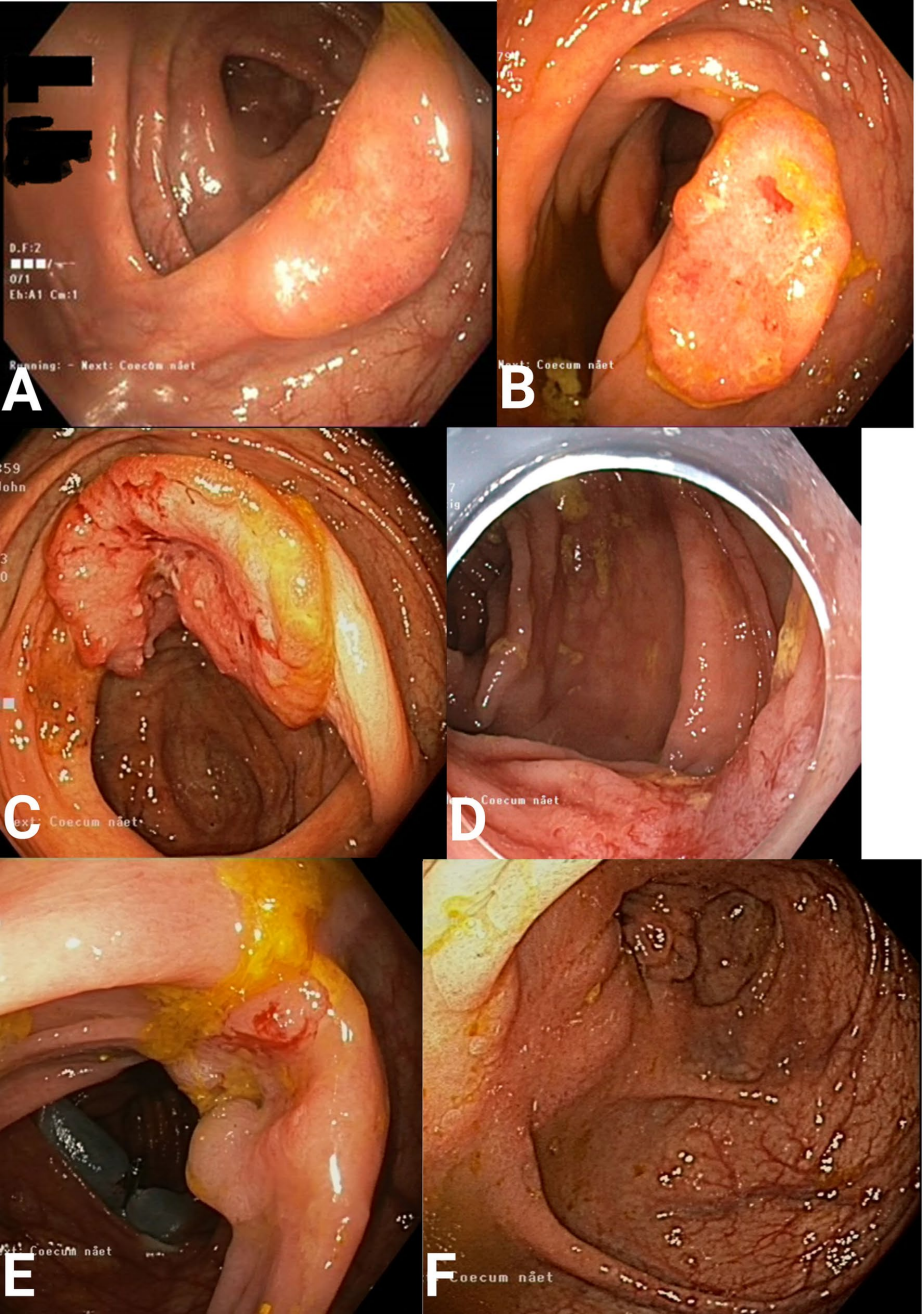
Preoperative endoscopic images of tumors resected by CELS. **A** Patient no.1. Biopsy showed an adenocarcinoma. **B** Patient no. 16. Biopsy showed high-grade neoplasia. **C** Patient no. 12. Biopsy showed an adenocarcinoma. **D** Patient no. 3. Biopsy showed high-grade neoplasia. **E** Patient no. 4. Biopsy showed an adenocarcinoma. **F** Patient no. 11. Biopsy showed high-grade neoplasia

**Fig. 3:**
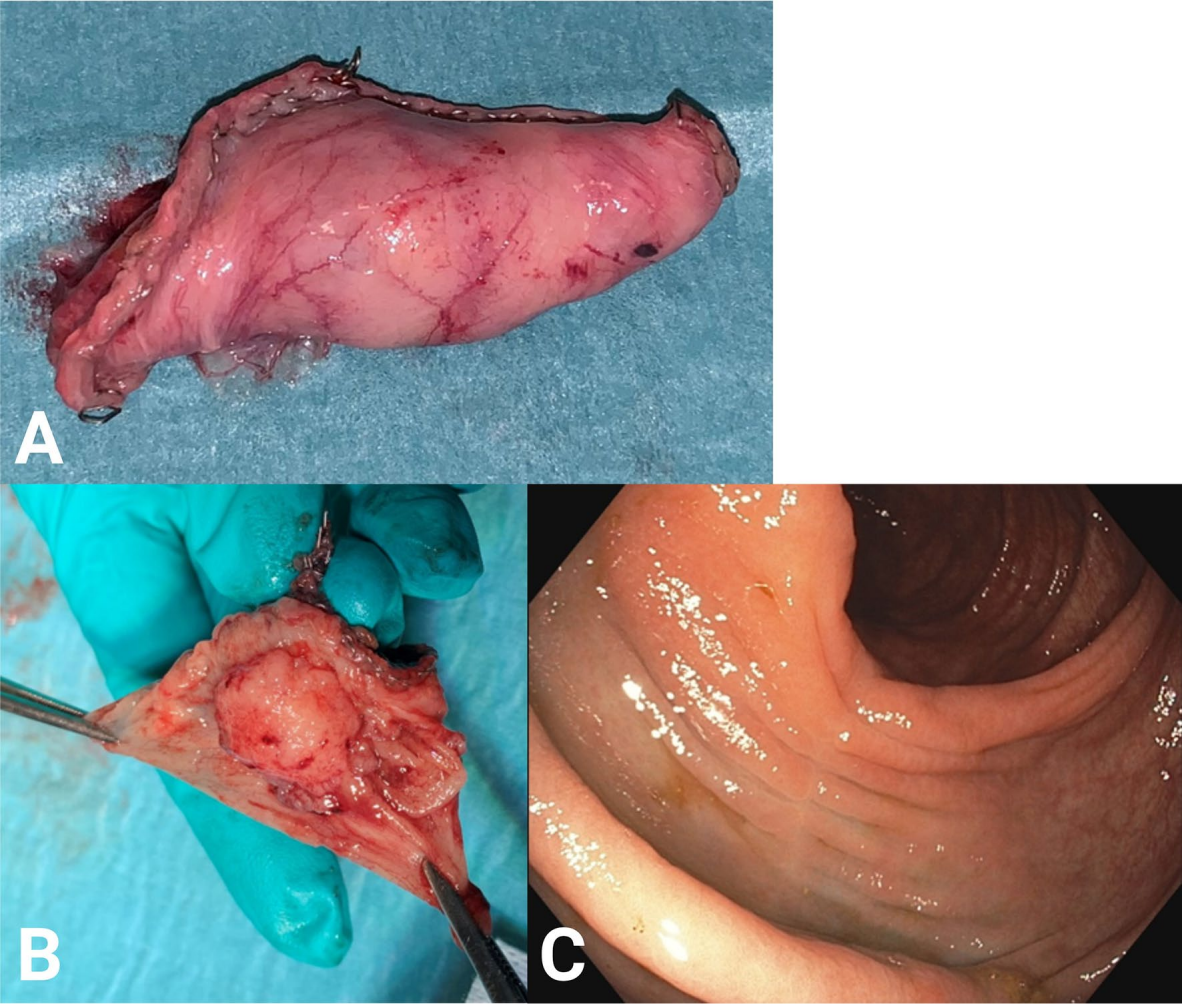
CELS resection specimen. **A** CELS specimen with serosal cover from patient no. 7. **B** Specimen cut open at the operating room with macroscopic free margins in patient no. 15. **C** Endoscopy 3 months after CELS showing scar formation in patient no. 5

**Fig. 4:**
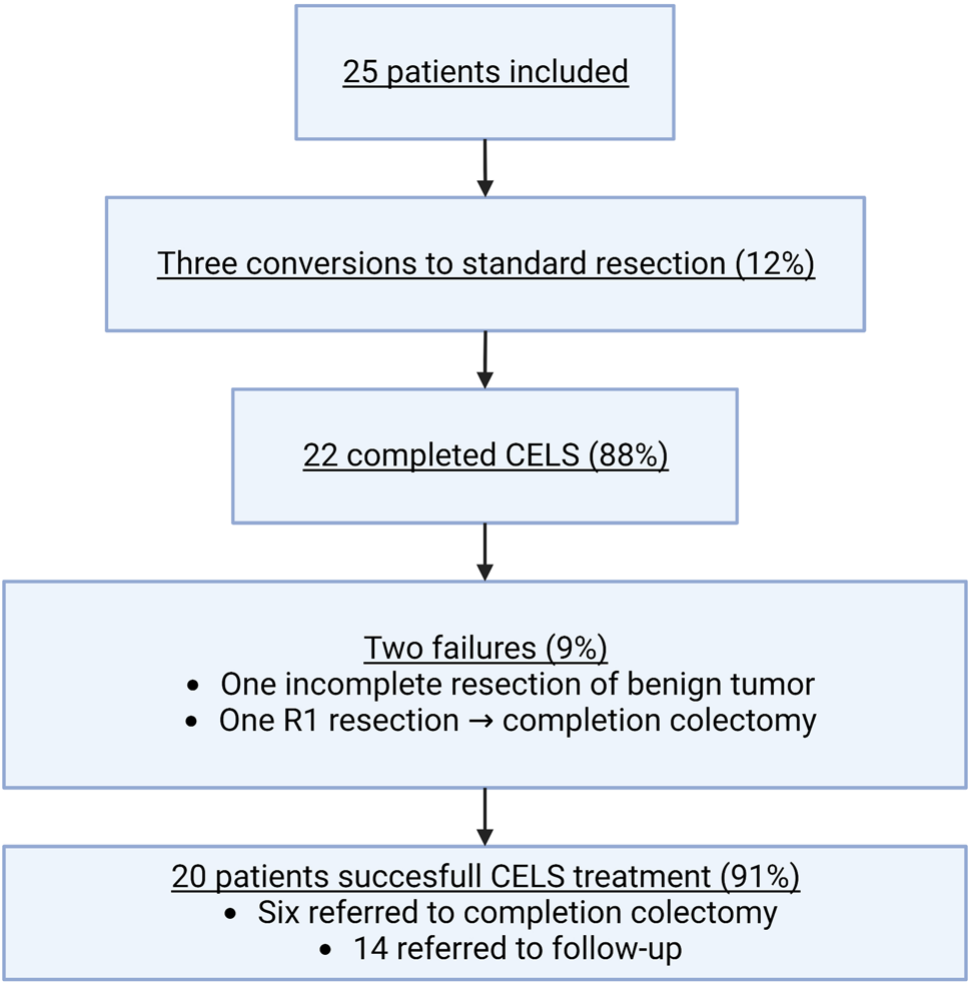
Study progression

In total, 25 patients (four females) were included during 2 years of enrollment. All patients invited for inclusion accepted. Median age was 77 years (63–90), 15 patients were classified as having cT1 and 10 as having cT2 tumors and median endoscopic evaluated tumor size was 20 mm (7–70 mm). Demographic data are shown in Table 1. A total of eight patients had a tumor in the cecum and 12 in the ascending colon, four patients had tumors in the transverse colon, and one patient had a tumor in the sigmoid colon. All tumors were either assessed suspected malignant based on the endoscopic appearance or with biopsy verified malignancy (Fig. 2). Examples of tumor specimens and bowel wall after CELS are shown in Fig. 3A–C. Study progression is shown in Fig. 4.

**Table 1:** Patient demographics

Patient no.	Age	Sex	PS/ASA score	BMI	Tumor placement	Tumor size (endo)	Clinical T category	OP time (min)	LoS
1	71	Male	0/3	30.3	Transverse colon (oral)	30	T1	61	1
2	78	Male	1/2	29.5	Cecum	20	T2	95	1
3	63	Male	1/2	30	Ascending colon	20	T2	42	1
4	73	Male	1/2	24	Transverse colon (anal)	15	T2	52	1
5	76	Male	1/2	19	Ascending colon	7	T1	44	2
6	77	Male	1/2	23	Ascending colon	15	T1	*Converted*	
7	83	Male	3/3	28	Cecum	15	T2	74	1
8	79	Male	2/3	22	Cecum	10	T1	69	1
9	74	Male	1/3	22	Cecum	15	T1	91	2
10^a^	77	Male	1/3	27	Ascending colon	15	T1	104	1
11	74	Male	1/3	25	Ascending colon	20	T1	113	2
12	77	Male	1/3	27.8	Cecum	30	T2	39	5
13	74	Male	0/3	29	Transverse colon (anal)	25	T1	81	1
14^a^	86	Male	1/3	23.9	Ascending colon	20	T2	122	2
15	74	Male	2/3	20	Transverse colon (anal)	20	T1	78	1
16	67	Male	0/3	28	Ascending colon	30	T1	52	1
17	74	Male	1/3	30	Ascending colon	20	T1	*Converted*	
18	90	Female	2/3	24	Cecum	15	T1	90	7
19	85	Male	1/3	21	Cecum	30	T2	55	2
20	81	Male	2/3	31	Ascending colon	30	T2	*Converted*	
21	73	Female	2/3	23	Ascending colon	13	T1	35	1
22	80	Male	1/4	31	Ascending colon	40	T1	31	2
23	81	Female	2/3	21.9	Sigmoid colon	40	T2	129	3
24	83	Female	1/2	33	Ascending colon	70	T1	46	1
25	72	Male	2/3	35	Cecum	25	T2	120	2

*PS* performance status, *ASA* American Society of Anesthesiologists, *BMI* body mass index, *Tumor size—endo* tumor size at preoperative endoscopy in mm, *OP time mins* operating time in minutes, *Converted* converted perioperative to standard oncological colectomy, *LoS* length of stay in days

^a^Treatment failure (R1 resection)

### Intraoperative data

**Fig. 5:**
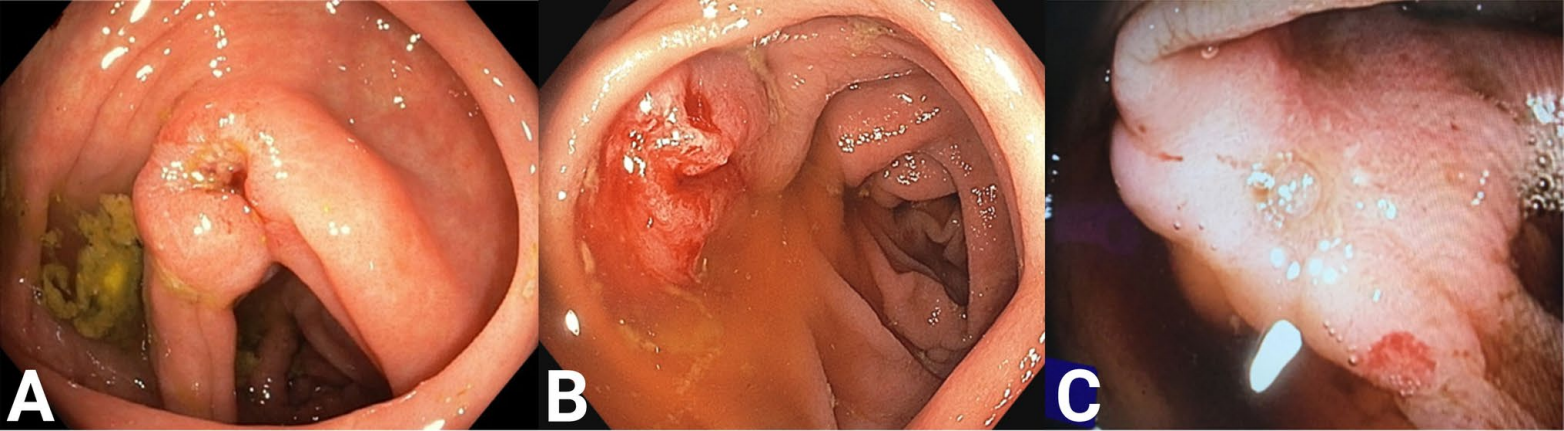
Intraoperative conversions. **A** Tumor described as 15 mm and ulcerated—cT1cN0. Located orally of the hepatic flexure in the ascending colon. Histopathological evaluation after surgery described a 10 mm pT2N0 tumor without histological risk factors. **B** A 20 mm polypoid tumor in the ascending colon near the hepatic flexure—cT1. The tumor was on the mesenteric side with diverticula around. Histopathological evaluation after surgery described a tumor with a 6 mm invasive focus, pT1sm1N0, without histological risk factors. **C** Tumor described as 4 cm in cecum. Tumor was perioperative assessed too large for local resection. Histopathological evaluation described a 12 mm pT3N0 tumor with perineural invasion. All cases where intraoperatively converted to standard colectomies

Twenty-two patients had CELS procedure performed and three patients were intraoperatively converted to standard hemicolectomy. Of these three patients, the first had an ulcerated 15 mm tumor in the mesenteric side of the ascending colon close to the hepatic flexure (Fig. 5A). The specimen from the CELS resection did not contain the tumor when it was cut open and standard colectomy was performed. The second patient had a 20 mm polypoid tumor in the ascending colon near the hepatic flexure. Despite mobilization it was assessed that local resection could not be performed (Fig. 5B). The third patient had a 40 mm tumor in the ascending colon (Fig. 5C). The tumor was assessed too large for local resection and a conventional resection was performed. All patients converted to standard colectomy had R0 resection.

For the 22 patients undergoing CELS, the median operation time was 71.5 min (31–129 min). Median number of laparoscopic stapler firings used for resection was 2.5 (1–5). Only 60 mm staplers were used. Mobilization of the colon was necessary in 15 cases. There were no intraoperative complications. Median length of stay (LoS) was 1 day (1–7 days).

### Complications

One patient developed a port site hematoma. The patient received two blood transfusions and a CT scan showed a port site hematoma with no sign of active bleeding. Three patients were re-admitted due to general discomfort, atrial fibrillation, and paralytic ileus, respectively, and all had short uncomplicated stays.

### CELS failures

**Fig. 6:**
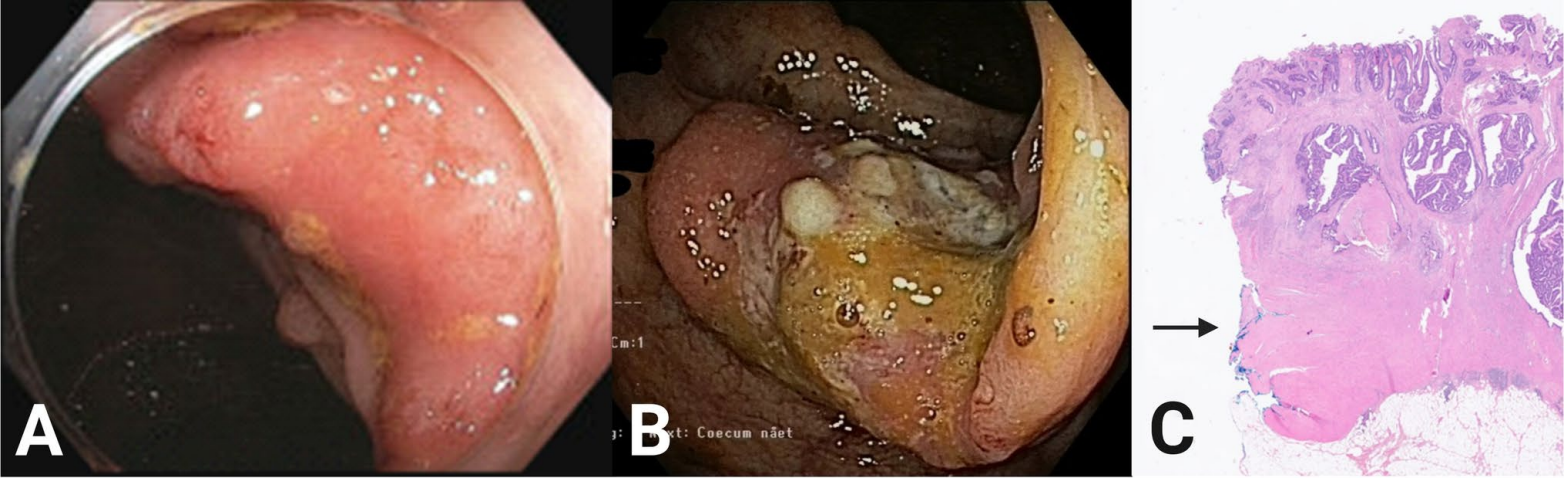
CELS failures. **A** Pedunculated polyp with non-lift sign. At macroscopic evaluation, it was difficult to define tumor demarcation. Histopathological assessment showed low-grade neoplasia with focal high-grade neoplasia. There was low-grade neoplasia in the resection margin after cutting the staple line off. Endoscopy after CELS showed relapse of the polyp and it was resected with endoscopic mucosal resection. The pathological assessment now showed free resection margins and the tumor was with low-grade neoplasia. **B** A 20 mm large tumor in the ascending colon. The CELS resection was performed as a sleeve resection including appendix. No macroscopic tumor tissue after the CELS. **C** Microscopic images of HE-stained tumor. The pathological assessment showed a 17 mm pT3 tumor with vascular invasion and without free margins (inked blue + arrow). Final histopathological assessment after completion colectomy showed a pT3N0 tumor with free margins (Color figure online)

Two patients had CELS failures due to incomplete resections. The first patient had a pedunculated tumor in the ascending colon. Histopathology showed an adenoma with a small focus of high-grade neoplasia, and with low-grade neoplasia in the resection margin (Fig. 6A). The second patient was an 86-year-old male patient with a tumor in the ascending colon near the ileocecal valve, assessed to be 20 mm at the preoperative endoscopy (Fig. 6B, C). The patient suffered from severe chronic obstructive pulmonary disease, atrial fibrillation, aortic stenosis, and was newly diagnosed with a marginal zone lymphoma in the lungs (ASA 3, PS 2). CELS was performed with no visible residual tumor in the bowel after local resection. However, at histopathological evaluation the resection margin was involved. The patient did not wish to undergo completion colectomy initially and a follow-up endoscopy 3 months later showed a local relapse. The patient had a completion colectomy performed subsequently. The resection specimen from the completion colectomy revealed a pT3N0 tumor. The patient died 15 months later due to comorbidities. The external safety committee did not find it necessary to terminate the study on the basis of these cases.

There were no cases of failure due to severe complications or death within 30 or 90 days after CELS.

### Histopathological evaluation

The pathological evaluation of the 22 patients that underwent CELS, categorized six patients with a pT1 tumor, eight with pT2, five with pT3, and three patients as having adenomas.

In total, 14 patients had histopathological high-risk factors present. Seven patients were not regarded fit for completion colectomy despite high-risk factors. Six patients were referred to completion colectomy besides the patient with incomplete resection described in the previous section (Table 2). None of the six patients had residual tumor or lymph node metastases.

**Table 2:** Histopathological characteristics of resected tumors

Patient no.	Pathological stage	Tumor size (mm)	Histologic subtype	Venous invasion	Perineural invasion	Lymphatic invasion	Budding	Deficient MMR	Radicality (R0/R1)
1	T2	10						+	R0
2	Adenoma	35	LGN						R0
3^a^	T3	19		+					R0
4	T2	20		+					R0
5	T2	27							R0
6	T2 N0	10							Converted
7	T1 Sm2	20				+			R0
8	T1 Sm1	6							R0
9^a^	T2	18			+	+			R0
10	Adenoma	18	HGN						*R1*
11	T1 Sm2	6		+					R0
12	T2	36		+					R0
13	T2	8		+	+				R0
14^a^	T3	17		+					*R1*
15	T1 Sm3	12	Mucinous					+	R0
16^a^	T2	16	Poorly diff			+	Bd3	+	R0
17	T1 sm1 N0	6							Converted
18	T3	25	Poorly diff	+		+	Bd2		R0
19	T1 Sm3	9							R0
20	T3 N0	12			+			+	Converted
21	T2	22						+	R0
22^a^	T1 Sm3	24		+	+			+	R0
23	Adenoma	21	HGN						R0
24^a^	T3	14	Mucinous					+	R0
25^a^	T3	35	Poorly diff	+		+	Bd3		R0

*LGN* low-grade neoplasia, *HGN* high-grade neoplasia, *Poorly diff.* poorly differentiated adenocarcinoma, *Deficient MMR* deficient mismatch repair protein on immunohistochemistry staining

^a^Completion colectomy

### Follow-up

Endoscopic follow-up after 3 months showed no signs of residual tumor in the 12 patients treated successfully with CELS for malignant tumors and who were not referred to completion colectomy. One of the patients developed a liver metastasis that was diagnosed 1 year after the CELS. The primary surgery found a 20 mm pT1Sm2 tumor with lymphatic invasion. Due to comorbidity, the patient did not have completion colectomy performed and CT scan after 6 months did not show any sign of metastasis. The patient underwent stereotactic radiotherapy for the liver metastasis and follow-up CT scan 8 months after radiotherapy showed no sign of relapse. Two patients died during follow-up due to causes unrelated with CELS and one patient resigned from follow-up after 6 months due to old age.

## Discussion

We included 25 high-risk patients with early-stage colon cancer for CELS resection. The procedure was abandoned in three cases. Two patients had treatment failure due to incomplete resection. Six patients underwent completion colectomy due to high-risk factors for LNM.

Selecting patients for organ-sparing surgery for early-stage colon cancer requires proper patient selection taking into account the risk of LNM and the risk of adverse outcome related to standard surgical treatment. In the present study, we selected patients with clinical suspicion of early-stage colon cancer for local tumor resection. The included patients were all regarded as having a high risk of complications and increased short-term mortality after conventional oncological resections. To our knowledge, the clinical feasibility of local wedge resection for early-stage colon cancer has not been reported previously. Our results demonstrate that 20 out of 22 patients, had a free resection margin after local resection. The patients were vulnerable, comorbid and elderly, but despite this, the median LoS was 1 day.

The only reported randomized trial regarding CELS compared to hemicolectomy by Lascarides et al., demonstrated that CELS as an endoscopic mucosal resection combined with laparoscopy, resulted in shorter LoS compared to traditional right-sided hemicolectomy [14]. Although bowel-sparring surgery probably has a lower risk of complications compared to conventional oncological resection, it is yet to be shown. A retrospective cohort study by Golda et al. [15] compared CELS with laparoscopic segmental colectomy for complex benign polyps and found reduced morbidity in the CELS group. The main CELS technique in that study was laparoscopic wedge resection.

The criteria for the tumor being technically suitable for CELS was only based on two parameters: tumors not occupying more than 50% of the bowel lumen when insufflated and not involving the ileocecal valve. The patient with a cancer and R1 resection had a 2 cm tumor near the ileac valve. The resection was limited by this tumor placement. An older study by Yan et al. [16] used more specific criteria for CELS suitability including a decreased maximum of lesion size for tumors located near the ileocecal valve.

The secondary outcomes in our study were all related to patient selection. We did not predefine a limit for our secondary outcomes, e.g., set a maximum amount of months for the study to be completed, since this study is the first to describe the patient selection. Despite the nature of this study and the risk of undergoing completion colectomy, all patients invited for inclusion accepted.

We used the definitions of histopathological high-risk factors used in the treatment guideline for malignant polyps containing pT1 cancers with some modifications [17, 18]. Particularly, a deeper tumor growth corresponding to pT2 category was not considered as a high-risk factor. However, patients with mucinous tumors and perineural invasion were offered completion colectomy at follow-up if they were assessed fit for surgery. The role of mucinous component in colorectal cancer and the risk of LNM is also subject to debate. A Swedish cohort study on patients with pT1 colorectal cancer found mucinous component to be an independent risk factor for LNM [19]. We found it necessary to include these variables as well as high-risk factors to ensure proper patient treatment. The assessment of patients who were regarded not fit for surgery was based on an overall assessment of the risk of adverse outcomes including how the patient managed through the course of the CELS treatment.

As a consequence of nationwide implemented screening for colorectal cancer, the future patients are expected to present with early-stage colon cancer more often [20]. This underlines the importance and implications of local resection. In cases where the tumor is very small or has a very large adenoma component, biopsies might not show adenocarcinoma, and deciding if the lesion is a cancer relies on the appearance of the tumor. For these instances, CELS resection could be regarded as an excision biopsy to clarify if the tumor is invasive and facilitate a stepwise surgical approach. The risk of LNM is different for a pT2 and a pT3 tumor, yet the recommendation for resection is the same [2, 5]. The difference in risk should not only be related to oncological outcomes but also include a patient-centered approach. The increased risk of LNM might be more acceptable for an elderly, comorbid or frail patient, providing the elderly patient can go through a smaller surgical procedure and discharged soon after.

### Limitations

The patient selection based on clinical staging by thoracic-abdominal CT scan represents a limitation in the study, since it has been shown to be inaccurate to identify patients with UICC stage-1 disease [21, 22].

The procedure was already a routine procedure at the primary site, but two of the secondary sites had little or no experience. At the secondary sites, dedicated surgeons and endoscopists conducted all procedures, inevitably there is still a learning curve. However, the procedure was easily implemented and for colorectal surgeons a relatively easy technique, which has also been previously described [23].

The majority of patients included had right-sided tumors. This was not intentional and represents a limitation when applying the procedure to left-sided tumors.

The specimens are small and sometimes very difficult to orientate for the pathologist. The area of the specimen where the mesentery of the colon is resected represents a circumferential resection margin (CRM). As a T2 tumor might be < 1 mm from the mesentery of the bowel, it is important that a possible CRM is marked. If there is serosal cover, the resection is R0, but if not, the resection is regarded as R1 [24]. Our experience is that the evaluation of the specimen in the operating room by both surgeons and pathologists entails a better understanding of the orientation and possible CRM. The UK guideline [24] on how to handle the specimen in terms of pathological reporting mainly focuses on endoscopic resections. A specific guideline on how to handle wedge resections should be developed.

Due to the small amount of patients and short follow-up, overall recurrence rate is not assessed in the study.

In conclusion, this study showed that for a selected group of patients, a local resection of early colon tumors was feasible and safe. CELS could be a favorable option for patients with a low risk of LNM combined with a high risk of adverse outcomes. However, patient selection should be performed in the multidisciplinary setting, and based on a combination of radiological and endoscopic findings, histology features, and clinical evaluation of the individual patient.
